# Synergistic Application of Molecular Markers and Community-Based Microbial Source Tracking Methods for Identification of Fecal Pollution in River Water During Dry and Wet Seasons

**DOI:** 10.3389/fmicb.2021.660368

**Published:** 2021-06-14

**Authors:** Hongxia Liang, Zhisheng Yu, Bobo Wang, Fabrice Ndayisenga, Ruyin Liu, Hongxun Zhang, Gang Wu

**Affiliations:** ^1^College of Resources and Environment, University of Chinese Academy of Sciences, Beijing, China; ^2^RCEES-IMCAS-UCAS Joint-Lab of Microbial Technology for Environmental Science, Beijing, China; ^3^State Key Laboratory of Urban and Regional Ecology, Research Center for Eco-Environmental Sciences, University of Chinese Academy of Sciences, Beijing, China; ^4^University of Chinese Academy of Sciences, Beijing, China

**Keywords:** microbial source tracking, fecal pollution, molecular markers, FEAST program, real-time quantitative PCR, high-throughput sequencing, dry and wet seasons

## Abstract

It is important to track fecal sources from humans and animals that negatively influence the water quality of rural rivers and human health. In this study, microbial source tracking (MST) methods using molecular markers and the community-based FEAST (fast expectation–maximization microbial source tracking) program were synergistically applied to distinguish the fecal contributions of multiple sources in a rural river located in Beijing, China. The performance of eight markers were evaluated using 133 fecal samples based on real-time quantitative (qPCR) technique. Among them, six markers, including universal (BacUni), human-associated (HF183-1 and BacH), swine-associated (Pig-2-Bac), ruminant-associated (Rum-2-Bac), and avian-associated (AV4143) markers, performed well in the study. A total of 96 water samples from the river and outfalls showed a coordinated composition of fecal pollution, which revealed that outfall water might be a potential input of the Fsq River. In the FEAST program, bacterial 16S rRNA genes of 58 fecal and 12 water samples were sequenced to build the “source” library and “sink,” respectively. The relative contribution (<4.01% of sequence reads) of each source (i.e., human, swine, bovine, or sheep) was calculated based on simultaneous screening of the operational taxonomic units (OTUs) of sources and sinks, which indicated that community-based MST methods could be promising tools for identifying fecal sources from a more comprehensive perspective. Results of the qPCR assays indicated that fecal contamination from human was dominant during dry weather and that fecal sources from swine and ruminant were more prevalent in samples during the wet season than in those during the dry season, which were consistent with the findings predicted by the FEAST program using a very small sample size. Information from the study could be valuable for the development of improved regulation policies to reduce the levels of fecal contamination in rural rivers.

## Introduction

Fecal contamination of surface water has been recognized as one of the leading causes of the decline of water quality worldwide (Blanch et al., 2006; Reischer et al., 2013). Human and animal feces could be inputted directly or indirectly into freshwater by multiple-point or nonpoint source pathways such as wastewater discharge, uncontrolled discard of feces from humans and animals, and rainwater runoff (Converse et al., 2012; De Kwaadsteniet et al., 2013; Dubinsky et al., 2016; Ahmed et al., 2017). In particular, rainwater runoff was considered one of the major sources of fecal matter transported into the freshwater environment, especially during the wet season in rural regions (Chidamba and Korsten, 2015; Kostyla et al., 2015; Ahmed et al., 2016). Microbial contaminations from human and animal fecal matter are a leading cause of the deterioration of water quality, raising public health concerns (Lee et al., 2013; Reischer et al., 2013). It is important to identify the sources of microbial contamination for the development of regulatory policies to protect water quality and avoid threats to human health caused by potential pathogenic bacteria from feces.

Microbial source tracking (MST) methods can distinguish fecal matter from different host species (Kildare et al., 2007; Staley et al., 2018; Balleste et al., 2020). The initially developed library-dependent MST methods relied on microbial reference libraries such as the routine collecting, monitoring, and typing of many isolates; therefore, it was time-consuming and costly to identify the potential fecal sources (Moore et al., 2005; Stoeckel and Harwood, 2007). Conversely, library-independent MST methods, targeting specific gene fragments (e.g., 16S rRNA gene) or taxonomic groups of the fecal sources associated with specific hosts, are considered accurate tools for the detection of fecal contamination and could be applied in diverse geographic settings with less time and effort (Fong et al., 2005; Kildare et al., 2007; Harwood et al., 2014).

In particular, MST methods based on molecular markers using real-time quantitative PCR (qPCR) and community-based programs using high-throughput sequencing (HTS) data have recently emerged. Up to now, MST molecular markers have been developed to distinguish fecal contamination from various host species, including human (Kildare et al., 2007; Reischer et al., 2007), swine (Mieszkin et al., 2009), ruminant (Kildare et al., 2007), avian (Ohad et al., 2016), and others (Marti et al., 2013). Although qPCR assays using MST markers with high sensitivity and specificity can distinguish fecal contamination from different host sources, each marker can only detect a specific source of contamination. In recent years, community-based MST methods, including random forest classifier (Smith et al., 2010; Roguet et al., 2018), the SourceTracker program (Knights et al., 2011), and the FEAST (fast expectation–maximization microbial source tracking) program (Shenhav et al., 2019), have been utilized to estimate the relative contribution of microbial contamination of each source to freshwater. These tools can match the microbial community profiles between potential sources and sink samples using the operational taxonomic unit (OTU) dataset of the 16S ribosomal RNA (rRNA) gene. More specifically, FEAST exhibited stronger robustness and higher running speed than the random forest classifier and SourceTracker under the same conditions (Shenhav et al., 2019). It has also been reported that the program could simultaneously determine the relative contribution of different sources in the same sample (Chen et al., 2020; Kim et al., 2020). However, the accuracy of the FEAST program in tracking fecal pollution requires further verification because its research on the fecal inputs of river waters has been minimal.

The objectives of this study were to distinguish the sources of fecal microbes in a rural river and quantify their relative contributions to the microbial community of the river water. Given the high accuracy of the molecular markers and the ability of FEAST to identify contributions from multiple sources, both MST methods were concomitantly applied to distinguish the sources of fecal pollution in the Fsq River, located in Beijing, China, during the dry and wet seasons.

## Materials and Methods

### Study Area

The mainstream of the Fsq River, located in Beijing, China, spans 18.2 km and covers 58.8 km^2^. Fsq River, one of the main tributaries of the Wenyu River, plays an important role in providing recreational and landscape water for surrounding residents. The Fsq River flows through many villages in the studied area, where the villagers breed swine or bovine on a small or a large scale. The Fsq River has been reported to be severely polluted by various sources such as human and animal feces, industrial wastewater, and farmland and woodland deposits. The sewage from many villages on both sides of the river is almost discharged directly into the Fsq River, resulting in poor water quality and subsequently affecting the water quality of the Wenyu River (Qi and Chen, 2012). Feces from villagers are stored in septic tanks and regularly removed by professional companies. There are sewage treatment stations in villages near the Fsq River, but only a small number of stations are in operation. Therefore, domestic sewage without treatment is usually discharged into the river through drainage ditches. In addition, farms near the river are not equipped with treatment systems for animal waste. The piled-up manure is likely to be washed into the river by rainwater runoff during the wet season.

The wet season with frequent rainfall events in the city of Beijing where the Fsq River is located mainly occurs from July to September, whereas the dry season covers the remaining months with little or no rainfall. All outlets on both sides of the Fsq River are open drainage ditches for the discharge of rainwater and domestic sewage. Only domestic sewage flows into the river through the drainage ditches in the dry season, whereas mixtures of rainwater, domestic sewage, and animal feces are discharged into the Fsq River in the wet season.

### Sampling

#### Fecal Samples

A total of 184 individual fecal samples were collected from each of human (*n* = 28) and 12 animal host species, including swine (*n* = 20), canine (*n* = 6), equine (*n* = 11), donkey (*n* = 4), bovine (*n* = 14), sheep (*n* = 18), goat (*n* = 5), chicken (*n* = 12), duck (*n* = 10), goose (*n* = 10), pigeon (*n* = 9), and fish (*n* = 37) (Supplementary Table 1). All fecal samples were collected in sterile 50-ml polypropylene tubes, which were transported directly to the lab on ice and immediately kept in the laboratory at −80°C until DNA extraction.

#### Water Samples

**FIGURE 1:**
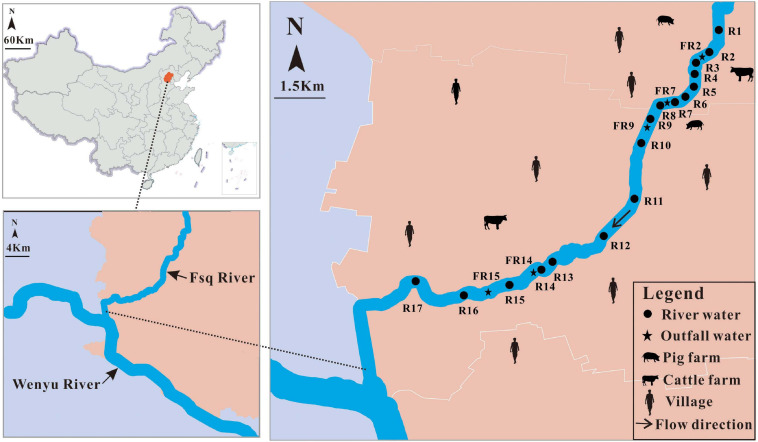
Overview of the sampling sites in Fsq River located in Beijing, China.

A total of 96 water samples were collected along the Fsq River in a downstream to upstream manner. River water samples (*n* = 80) were collected from 17 sites, R1 to R17, and outfall water samples (*n* = 16) were obtained from five sites, including FR2, FR7, FR9, FR14, and FR15 (Figure 1 and Supplementary Table 2). All water samples were collected from five sampling events, named BF, AF1, AF2, AF3, and AF4, between July and November 2019. There was no rainfall within 1 week before the occurrence of sampling event BF during the dry season, thus reflecting the pollution status of the river water without rain interference. Samples for sampling events AF1 to AF4 were obtained as early as possible after the start of rain events during the wet season. Water samples were collected in 1-L bottles using either rope-suspended samplers or sampling poles. All water samples were transported directly to the lab on ice and immediately filtered. Briefly, 1 L of each water sample was filtered through 47 mm × 0.22 μm nitrocellulose membrane (Millipore, Billerica, MA, United States) to retain microbial cells, and then filter papers were stored in the laboratory at −80°C as fecal samples.

### DNA Extraction and Sequencing

Genomic DNA was extracted from fecal (180–220 mg) and water (filter papers) samples using the DNeasy PowerSoil Kit (QIAGEN, Hilden, Germany) used in the Earth Microbiome Project and then purified using the OneStep^TM^ PCR Inhibitor Removal Kit (Zymo Research Corp., Irvine, CA, United States) following the manufacturer’s instructions. The purity and concentration of genomic DNA were measured with a NaNodrop 2000 spectrophotometer (Thermo Fisher Technologies, Foster City, CA, United States). Purified DNA extracts were sent to Majorbio Bio-Pharm Technology Co., Ltd. (Shanghai, China) for sequencing of the bacterial V3–V4 region of the 16S rRNA gene on an Illumina MiSeq platform using forward primer 338F (5′-ACTCCTACGGGAGGCAGCAG-3′) and reverse primer 806R (5′-GGACTACHVGGGTWTCTAAT-3′) (Mori et al., 2014). The reads obtained in the current study have been deposited in the SRA database (NCBI) under the BioProject accession number PRJNA713417, with Biosample accession numbers SAMN18274560–SAMN18274616 and SAMN18252387–SAMN18252398. Besides, the sequence data of one bovine feces have been deposited in the SRA database (NCBI) under the BioProject accession number PRJNA392724, with biosample number SAMN07305415.

### Selection of Markers and Community-Based Programs

For qPCR assays, eight molecular markers that performed well in previous reports were selected in the study, including universal marker BacUni, four human-associated markers (HF183-1, HF183-2, BacH, and BacHum), swine-associated marker Pig-2-Bac, ruminant-associated marker Rum-2-Bac, and avian-associated marker AV4143 (Supplementary Table 3). The universal marker BacUni has demonstrated a 100% positive rate against human and animal fecal samples in several countries (Kildare et al., 2007; Nshimyimana et al., 2017), including China (Liang et al., 2020). Four human-associated markers—HF183-1, BacH, HF183-2, and BacHum—were recommended in the studies conducted in China (Vadde et al., 2019; Zhang et al., 2020) and other countries (Reischer et al., 2013; Odagiri et al., 2015; Haramoto and Osada, 2018). The high prevalence rates of Pig-2-Bac and Rum-2-Bac in target host species were also reported in China (He et al., 2016; Xu et al., 2020; Zhang et al., 2020) and in an evaluation study of 27 labs (Boehm et al., 2013). The avian-associated marker AV4143 was modified in our previous study and showed high sensitivity and specificity against fecal samples from China (Liang et al., 2020).

The relative contributions of microbes from potential fecal sources to sink samples were estimated using FEAST, a highly efficient expectation/maximization-based program (Shenhav et al., 2019). A closed OTU dataset was generated consisting of 70 regionally specific source and sink samples. The OTU dataset of individual fecal samples (*n* = 58) was obtained to develop the “source” library (Supplementary Tables 1, 4). The OTU dataset of river water samples (*n* = 12) collected from locations R1 to R6 during the dry (BF) and wet (AF3) seasons was designated “sink.” The FEAST program was run under the “FEAST example Multiple sinks” script to screen fecal sources to sink samples (Supplementary Table 4). For each sink, five independent runs were carried out in R version 3.6.3 (Vienna, Austria) to reduce the effect of false predictions, as in a previous study (Henry et al., 2016).

In the qPCR assays, the performances of eight selected markers were first evaluated in 133 fecal samples from 13 host species (Supplementary Table 1). Subsequently, only markers that performed well were used to track the sources of all 96 water samples (Supplementary Table 2). In the FEAST program, 16S rRNA genes of fecal (*n* = 58) and water (*n* = 12) samples (Supplementary Tables 1, 4) were sequenced to build the “source” library and “sink.” Compared with a foreign source library, a local source library could efficiently distinguish fecal sources in the sink samples (Staley et al., 2018), and thus the fecal samples used to build the “source” library were collected from China in this study. In addition, fecal sources of the water samples were identified with MST markers, and then only the identified fecal sources were contained in the “source” library to obtain more reliable prediction results by the FEAST program (Brown et al., 2019).

### qPCR Assays

All the MST markers in this study were monitored on an ABI 7500 real-time qPCR system (Applied Biosystems, Foster City, CA, United States). The optimized reaction mixture (20 μl) was composed of 10 μl 2 × Premix Ex Taq (Probe qPCR; Takara Bio, Otsu, Japan), 0.2 μl Rox Reference Dye II (50×; Takara Bio, Otsu, Japan), primers and probes at the final concentrations in the mix shown in Supplementary Table 3, 2 μl template DNA, and nuclease-free water to a final volume of 20 μl. The TaqMan PCR program was initiated at 50°C for 2 min and 95°C for 30 s, followed by 40 cycles of denaturation at 95°C for 5 s and annealing/extension at 60°C for 1 min. All reactions, including those of the samples tested, standards (Supplementary Table 5), and no-template controls, were performed in triplicate using MicroAmp Optical 96-well reaction plates.

The standard curve for each of eight TaqMan qPCR assays (Supplementary Table 3) was established using six 10-fold serial dilutions (10^3^–10^8^ gene copies per reaction) of plasmid standards (Supplementary Table 5) containing the target gene sequences. The amplification efficiency, limit of detection (LOD), and limit of quantification (LOQ) were measured based on the generated standard curves (Supplementary Table 6). The amplification efficiency (*E*) was calculated according to the formula: *E* = 10^(1/–slope)^ − 1 (Bustin et al., 2009). LOD was the lowest number of gene copies detected in the target host samples. LOQ was considered the lowest concentration within the linear range of quantification. Once the LOD and LOQ were confirmed, the absence of markers in samples can be divided into ND (not detected, no amplification), DNQ (detected but not quantifiable, LOQ < *C*_*t*_ < LOD), and ROQ (detected within the range of quantification, *C*_*t*_ < LOQ). To ensure reproducibility, the standards of each marker were tested in different plates (Supplementary Table 7), as described in previous studies (Nshimyimana et al., 2017; Zhang et al., 2020). The performances of the universal and specific markers were evaluated according to sensitivity (*R*), specificity (*S*), and accuracy (*A*) (Kildare et al., 2007; Odagiri et al., 2015). They were defined as follows: *R* = TP/(TP + FN) × 100%, *S* = TN/(TN + FP) × 100%, and *A* = (TP + TN)/(TP + FP + TN + FN) × 100%, where TP and FN represent true positive and false negative, respectively, in the target host samples and TN and FP are true negative and false positive, respectively, in the non-target host samples.

Cross-reactivity was defined as the condition at which the marker was found to be positive in the non-target samples. A “25th/75th” metric was applied to determine the classification of cross-reactivity for each marker in the non-target host samples (i.e., 25th/75th metric = 25th percentile_*target*_ - 75th percentile_*non–target*_) (Reischer et al., 2013). Accordingly, the samples were classified into four groups (Zhang et al., 2020) as follows: no cross-reactivity (NCR), when the marker did not show any positive signal in the non-target samples; weak cross-reactivity (WCR), when the “25th/75th metric” > 0; moderate cross-reactivity (MCR), when the “25th/75th metric” <0; and strong cross-reactivity (SCR), when either the disparity between the mean gene copies of the target and non-target samples was below one order of magnitude or the number of mean gene copies of the non-target samples was higher than that in the target samples.

### Bioinformatics and Statistical Analyses

The raw 16S rRNA gene sequences were quality filtered using Fastp software (v.0.20.0) (Chen et al., 2018). Reads were filtered to remove adapters and trimmed to remove any terminal stretches of bases at or below Q30. FLASH software (v.1.2.11) was used to merge into a single read of sequences that passed the quality control (Magoc and Salzberg, 2011). Chimeric sequences were checked and removed using USEARCH v.7.0 (Edgar, 2010). Sequences with ≥97% similarity were assigned to the same OTU and the representative sequence was screened for each OTU using UPARSE v.7.0 (Edgar, 2013). The sequences were further filtered to remove OTUs that accounted for less than 0.005% of the total sequence counts (Bokulich et al., 2013). Representative sequences were used to annotate taxonomic information against the Silva database (release 138) using the RDP Classifier (v.2.2) on the QIIME platform 1.9.0 based on the Bayesian algorithm (Caporaso et al., 2010). Statistical analyses and data visualization were carried out using SPSS version 25.0 (SPSS Inc., Chicago, IL, United States) and R version 3.6.3 (Vienna, Austria), respectively.

## Results

### Selection of MST Markers Used for Water Samples

#### Prevalence of MST Markers in Human and Animal Fecal Samples

We used 133 individual fecal samples to evaluate the sensitivity, specificity, and accuracy of eight MST markers using TaqMan qPCR. Based on this, we only selected the markers effective at detecting the fecal pollution of water samples. Samples identified as ND were considered negative, whereas those identified as DNQ and ROQ were treated as positive in this study.

**FIGURE 2:**
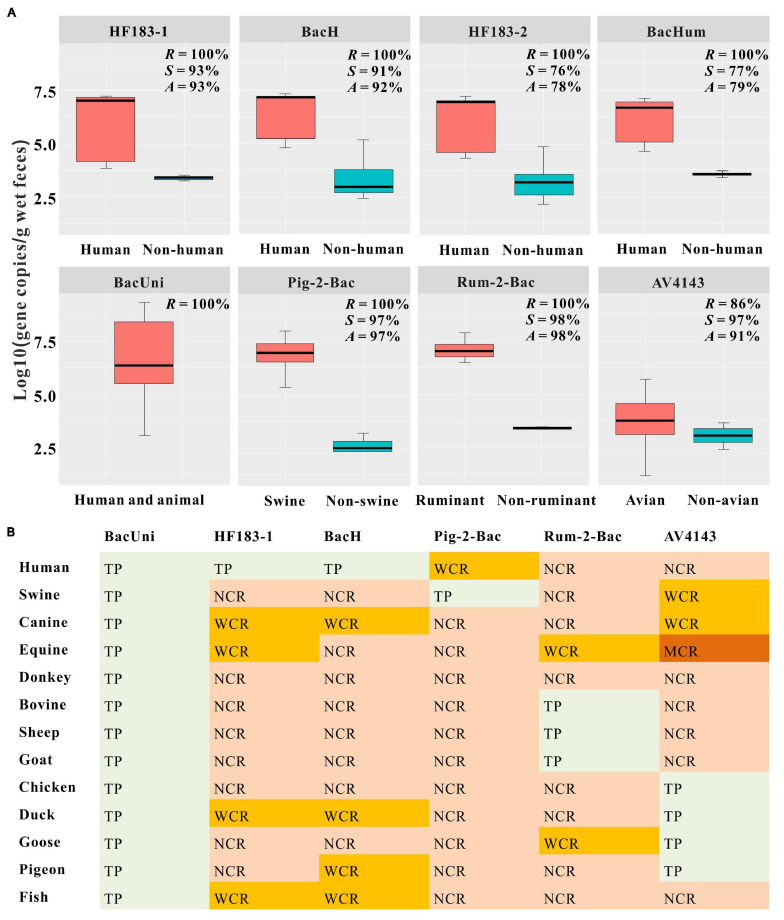
Concentrations **(A)** and classification of cross-reactivity. **(B)** for each tested marker. **(A)** Prevalence and concentrations of the universal marker BacUni, human-associated markers (HF183-1, BacH, HF183-2, and BacHum), pig-associated marker (Pig-2-Bac), ruminant-associated marker (Rum-2-Bac), and avian-associated marker (AV4143) in fecal samples from human, swine, canine, equine, donkey, ruminant (bovine, sheep, and goat), avian (chicken, duck, goose, and pigeon), and fish. Letters *R*, *S*, and *A* represent the sensitivity, specificity, and accuracy of each marker, respectively. Each box plot shows the median, upper, and lower quartiles spanning the maximum and minimum observations. Log-transformed gene copies of negative results were treated as 0 value. Only positive results within the limit of detection (LOD) were displayed in the graph. **(B)** Classification of cross-reactivity in the non-target samples for each microbial source tracking (MST) marker used to detect fecal pollution from environmental samples. The results were colored based on the following criteria: TP (true positive); NCR (no cross-reactivity), no false-positive signal was amplified; WCR (weak cross-reactivity), the “25th/75th metric” >0; MCR (moderate cross-reactivity), the “25th/75th metric” <0; SCR (strong cross-reactivity), the disparity of the mean gene copies between the non-target and target hosts is less than one order of magnitude or the mean gene copies of the non-target samples are higher than those in the target samples.

We found that the universal marker BacUni exhibited 100% sensitivity because it was detected in all fecal samples from humans (*n* = 13) and animals (*n* = 120) (Figure 2 and Supplementary Table 8). In the case of the four human-associated markers, we observed that both HF183-1 and BacH performed well, with ≥92% accuracy, followed by HF183-2 and BacHum, with ≤77% accuracy. The sensitivity of each of HF183-1, BacH, HF183-2, and BacHum was calculated as 100%, but their specificity values were 93%, 92%, 76%, and 77%, respectively. We also found that the markers Pig-2-Bac, Rum-2-Bac, and AV4143 displayed ≥91% accuracy. More specifically, we noticed that the three markers were not only present in the fecal samples of 100% swine, 100% ruminant (i.e., bovine, sheep, and goat), and 86% avian (i.e., chicken, duck, goose, and pigeon), respectively, but also demonstrated ≥97% specificity against the non-target host samples (Figure 2 and Supplementary Table 8).

#### Concentrations of MST Markers in Human and Animal Fecal Samples

Using standards in each qPCR assay, we observed that the amplification efficiencies ranged from 87.1% to 101.9% (Supplementary Table 6), meeting the requirements of the MIQE (minimum information for the publication of qPCR) guidelines (Bustin et al., 2009). The LOD and LOQ for each qPCR assay are also exhibited in Supplementary Table 6. Moreover, the inhibitors in the samples used in this study were found to have little effect on the qPCR assays, in accordance with our previous research (Liang et al., 2020). The credibility of the qPCR results was further proven by the ideal reproducibility of the qPCR assays on different reaction plates. We found that the mean coefficient of variability (CV) was less than 4.0% ± 0.1% for each standard between 10^3^ and 10^8^ gene copies/μl (Supplementary Table 7). Marker abundance was characterized as log10 (gene copies) per gram wet feces or log10 (gene copies) per 100 ml water, as discussed in previous studies (Kildare et al., 2007; Nshimyimana et al., 2017).

In general, we observed that the universal marker BacUni showed higher gene copy numbers than the specific markers in the target hosts. In particular, we found that the concentrations of BacUni were 6.70 ± 1.71 (mean ± standard deviation) in all the fecal samples tested from humans and animals (Figure 2A and Supplementary Table 8). Despite the presence of false-positive samples in each human-associated marker tested in this study, we noticed significant differences in the concentration of each marker between the fecal samples from humans (mean ± SD > 6.16 ± 1.4) and non-human hosts (mean ± SD < 3.59 ± 0.33; one-way ANOVA: *P* < 0.01) (Figure 2A and Supplementary Table 8). Likewise, approximately four orders of magnitude and significant differences were also observed for Pig-2-Bac and Rum-2-Bac between the target host (mean ± SD = 6.84 ± 0.84 and 7.02 ± 0.50) and the non-target host (mean ± SD = 2.66 ± 0.41 and 3.41 ± 0.06) fecal samples (one-way ANOVA: *P* < 0.01) (Figure 2A and Supplementary Table 8). In contrast, we noticed that the avian-associated marker AV4143 exhibited low concentrations (mean ± SD = 3.81 ± 1.09) in the target samples compared with the other host-associated markers tested in this study. The performances of HF183-2 and BacHum were poor (Figure 2A and Supplementary Table 8); thus, we did not further classify the cross-reactivity of these two markers. We consecutively divided the cross-reactivities of HF183-1, BacH, Pig-2-Bac, and Rum-2-Bac in the non-target host fecal samples into NCRs or WCRs (Figure 2B and Supplementary Table 9). In the AV4143 assay, we found that only one positive sample from equine was classified as MCR, whereas the others were NCRs or WCRs (Figure 2B and Supplementary Table 9).

#### Screening of MST Markers Used for Water Samples

Based on the results of the qPCR assays, we selected six markers, including the universal marker BacUni, the human-associated markers HF183-1 and BacH, swine-associated marker Pig-2-Bac, the ruminant-associated marker Rum-2-Bac, and the avian-associated marker AV4143, to distinguish fecal pollution in water samples. Each of these markers met the 80% specificity and sensitivity benchmarks used in a previous report (Boehm et al., 2013) and the guideline document of the United States Environmental Protection Agency (USEPA) (U.S. Environmental Protection Agency, 2005) when DNQ was considered positive (Figure 2A and Supplementary Table 8). In addition, we found that the cross-reactivity for each selected marker ranged only from 2% to 9% in the non-target hosts, with almost all of them being grouped into WCR or NCR, which indicated that there was no or little effect on the qPCR assays (Figure 2B and Supplementary Table 9).

### Application of MST Markers Selected in Water Samples

We analyzed all 96 water samples, including river water (*n* = 80) and outfall water (*n* = 16), using the six aforementioned markers selected from the previous step. We accordingly found that all the water samples from the river and outfalls consistently showed 100% positive signals for total Bacteroidales (BacUni; Table 1). In particular, we detected that the human-associated markers (60% in HF183-1 and 69% in BacH) exhibited the highest prevalence in all the river water samples tested, followed by the ruminant-associated marker Rum-2-Bac (29%) and finally the swine-associated marker Pig-2-Bac (3%) and the avian-associated marker AV4143 (0%; Table 1). The positive rates of markers HF183-1 and BacH were demonstrated to be higher in river samples during the dry season (87% and 93%, respectively) than those from the wet season (60% and 69%, respectively). However, we observed the opposite trend in the detection rates for markers Pig-2-Bac (0% and 3%) and Rum-2-Bac (7% and 34%) in the samples collected from the dry and wet seasons, respectively (Table 1). A similar pollution trend was also observed in the outfall water samples. More specifically, we found that markers HF183-1 and BacH showed the highest positive rates (38 and 56%), followed by Rum-2-Bac (13%) and finally Pig-2-Bac (6%) and AV4143 (0%) in the outfall water samples (Table 1 and Supplementary Table 10).

**TABLE 1:** Prevalence of the microbial source tracking (MST) markers in the water samples collected from the river and outfalls.

Sample	Season	Sampling events	No. of samples	Positive ratios					
				Total (%)	Human (%)		Swine (%)	Ruminant (%)	Avian (%)
				BacUni	HF183-1	BacH	Pig-2-Bac	Rum-2-Bac	AV4143
River	Dry	BF	15	100	87	93	0	7	0
	Wet	AF	65	100	60	69	3	34	0
	Total		80	100	60	69	3	29	0
Outfall	Dry	BF	1	100	100	100	0	0	0
	Wet	AF	15	100	33	53	7	13	0
	Total		16	100	38	56	6	13	0

**FIGURE 3:**
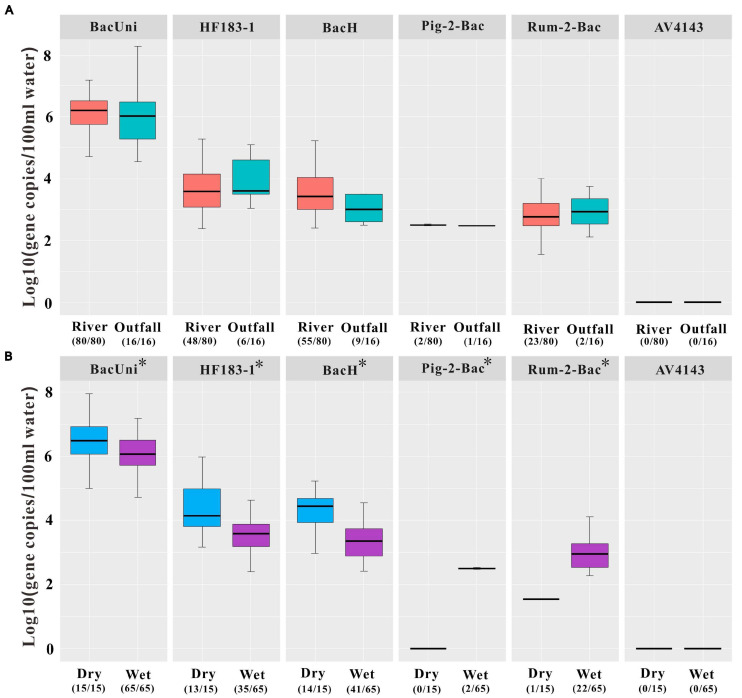
Concentrations of the universal marker BacUni, the human-associated markers HF183-1 and BacH, the swine-associated marker Pig-2-Bac, the ruminant-associated marker Rum-2-Bac, and the avian-associated marker AV4143 in environmental samples, including **(A)** all river water and outfall water samples and **(B)** river water samples during the dry and wet seasons. *Asterisk* indicates that statistically significant differences were observed in the concentrations of the markers in the river water samples between the dry and wet seasons by independent-samples *t*-test at the 0.05 level of significance. *Numbers in parentheses* represent the sample number within the limit of detection (LOD)/total samples number tested. Each box plot shows the median, upper, and lower quartiles spanning the maximum and minimum observations. Log-transformed gene copies of negative results were treated as 0 value. Only positive results within the limit of quantification (LOQ) were displayed in the graph.

We further observed a strong correlation of the mean concentrations of the tested markers between the positive river and outfall water samples (Spearman: *R* = 0.85, *P* < 0.01). Signals of HF183-1 and BacH were detected in both the river (mean ± SD = 3.76 ± 0.72 and 3.61 ± 0.75) and outfall (mean ± SD = 4.03 ± 0.78 and 3.56 ± 1.16) water samples (Figure 3A and Supplementary Table 10). Likewise, Bacteroidales from ruminant feces (Rum-2-Bac) were quantified at the concentrations of 2.97 ± 0.65 in 23 of the 80 river water samples and 2.93 ± 1.16 in two of the 16 outfall water samples (Figure 3A and Supplementary Table 10). Signals of Pig-2-Bac were detected only in the river water samples (mean ± SD = 2.50 ± 0.05) collected at locations R2 (2.53) and R3 (2.47) and in the outfall water sample collected at location FR9 (2.47) during the wet season (Figure 1), which were adjacent to a large-scale pig farm in the village nearby (Figures 1, 3A and Supplementary Table 10). No amplification of AV4143 was found in the outlet water at each sampling event (Figure 3A).

There were significant statistical differences in the concentrations of the markers BacUni, HF183-1, BacH, Pig-2-Bac, and Rum-2-Bac between the river water samples collected during the dry and wet seasons (independent-samples *t*-test: *P* < 0.05; Figure 3B). In particular, we found that the concentrations of total and human-specific Bacteroidales (i.e., BacUni, HF183-1, and BacH) in the river water samples were higher in dry weather (mean ± SD = 6.53 ± 0.84, 4.34 ± 0.82, and 4.25 ± 0.90, respectively) than those in the wet season (mean ± SD = 6.13 ± 0.60, 3.55 ± 0.56, and 3.40 ± 0.56, respectively) (Figure 3B and Supplementary Table 10). On the contrary, higher levels of Pig-2-Bac and Rum-2-Bac were observed in the river water samples collected during the wet season (mean ± SD = 2.50 ± 0.05 and 2.97 ± 0.65) than during the dry season (0 and 1.54) (Figure 3B and Supplementary Table 10).

### High-Throughput Sequencing Analyses

A total of 58 fecal samples and 12 river water samples were sequenced in this study (Supplementary Table 4). The average sequence numbers from the water and fecal samples were 48,288 and 36,515, respectively (Supplementary Table 11). We noticed that the microbial community showed a lower average *α* diversity in the fecal samples than in the river water samples (Supplementary able 11). Feces had an average Shannon index of 4.30 ± 0.93 and 3,666 OTUs that clustered at 97% similarity, whereas freshwater samples had an average Shannon index of 4.92 ± 1.18 and 5,561 OTUs clustering at 97% similarity (Supplementary Table 11).

**FIGURE 4:**
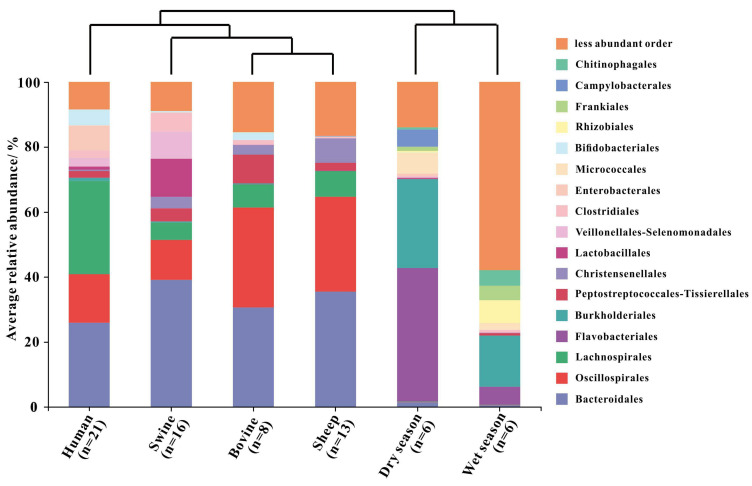
Hierarchical clustering tree based on Bray–Curtis and the relative abundance of the bacterial community on the order level in the fecal samples (human, swine, bovine, and sheep) and river water samples collected from the dry and wet seasons. Only orders with a relative abundance higher than 4% were represented in the legend.

Fecal and river water samples were clustered separately based on the analysis of the hierarchical clustering tree of the Bray–Curtis dissimilarity matrix (Figure 4 and Supplementary Figure 1). Differences in the microbial communities between the fecal and water samples were demonstrated according to the presence and abundance of specific bacteria. In the bacterial community of the fecal samples, four of 17 orders accounted for 56.77%–72.61% of the sequence data across human and animal fecal samples. The four orders were Bacteroidales (25.92%–39.12%), Oscillospirales (12.23%–30.72%), Lachnospirales (5.42%–28.71%), and Peptostreptococcales–Tissierellales (2.10%–8.83%) (Figure 4 and Supplementary Table 12). The orders Flavobacteriales (41.06% and 5.53%) and Burkholderiales (27.34% and 15.74%) were found to be the most common taxa in the water samples during the dry and wet seasons, respectively (Figure 4 and Supplementary Table 12). In addition, we observed that each of the nine orders showed different relative abundance among water and each kind of host fecal sample, including Bacteroidales (0.54%–39.12%), Oscillospirales (0.03%–30.72%), Lachnospirales (0.08%–28.71%), Burkholderiales (0.01%–27.34%), Peptostreptococcales–Tissierellales (0.09%–8.83%), Christensenellales (0.01%–7.53%), Lactobacillales (0.02%–11.73%), Clostridiales (0.20%–5.77%), and Enterobacterales (0.02%–7.79%).

**FIGURE 5:**
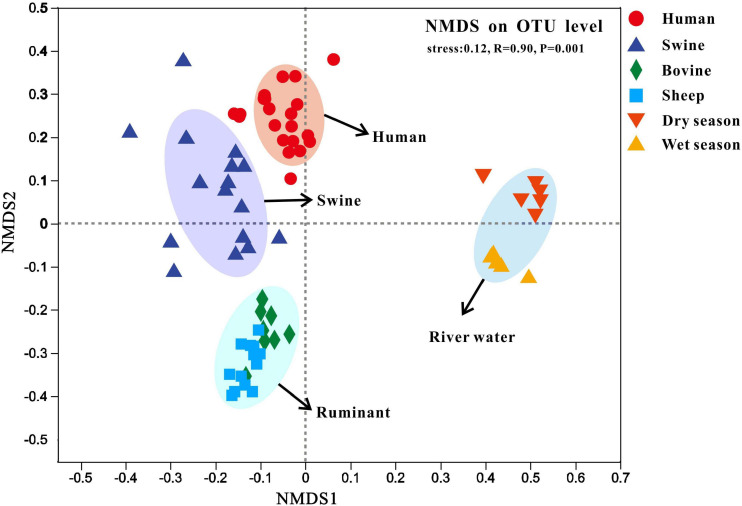
Non-metric multidimensional scaling (NMDS) analysis of the Bray-Curtis dissimilarities among sample bacterial communities on the operational taxonomic unit (OTU) level. Fecal samples were collected from human, swine, bovine, and sheep. River samples were collected from the Fsq River during the dry and wet seasons.

We also performed non-metric multidimensional scaling (NMDS) analyses based on the Bray–Curtis dissimilarities between the fecal (i.e., human, swine, bovine, and sheep) and river water samples collected during the dry and wet seasons (Figure 5). Similar to the results of hierarchical clustering, the fecal and river water samples were clustered independently with a statistically significant difference (stress = 0.123, *R* = 0.91, *P* < 0.01). Moreover, we found that the fecal samples from different host species were also clustered independently, whereas those of bovine and sheep belonging to ruminants were classified as the same cluster.

### Fecal Sources Determined by FEAST Analyses

**FIGURE 6:**
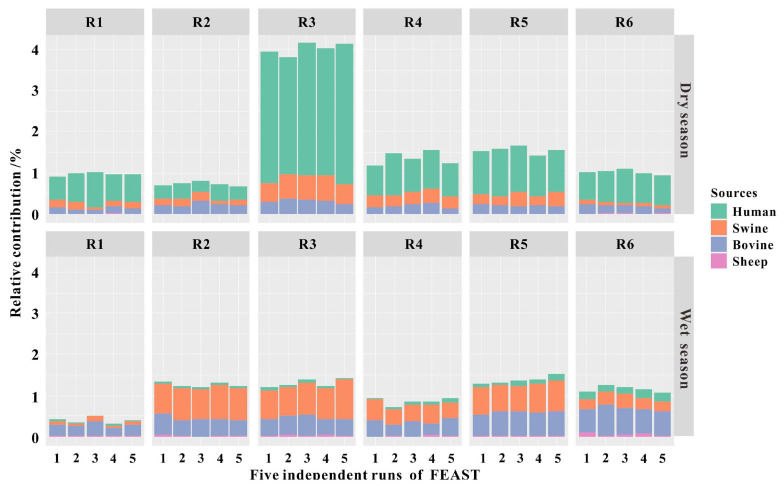
Fast expectation–maximization microbial source tracking (FEAST) analyses showing the relative contributions of fecal pollution sources in river water collected from R1 to R6 during the dry and wet seasons. A *single bar* represents the relative contribution in an independent FEAST run using the same script. FEAST was run in five independent runs on the same script.

As fecal pollution from hosts of humans, swine, and ruminants (i.e., bovine and sheep) has been identified as a source in the Fsq River based on the results of the qPCR assays, we sequenced the fecal samples (*n* = 58) from these host species to create a “source” library. The sink was built using the 16S rRNA gene sequences of the river water samples (*n* = 12) collected from R1 to R6, six different locations in the Fsq River, during the dry and wet seasons. Subsequently, we ran a total of five independent analyses on the OTU level taxa tables by the FEAST program to predict the proportions of fecal sources in the river water samples. We accordingly observed that bacterial signatures resembling fecal sources represented low levels of water contamination (<4.01% of sequence reads) in the bacterial communities of the river samples (Figure 6 and Table 2). Potentially unknown sources accounted for a high level (>95.99% of sequence reads), owing to the complex community composition (Table 2).

**TABLE 2:** Relative contributions of potential fecal sources based on FEAST analyses in river water samples.

Season	Sink	Relative contribution of source (%)				
		Human	Swine	Bovine	Sheep	Unknown
Dry	R1	**0.69**^*a*^	0.15	0.13	0.01	99.02
	R2	**0.34**	0.15	0.24	0	99.27
	R3	**3.15**	0.55	0.31	0	95.99
	R4	**0.85**	0.3	0.2	0	98.65
	R5	**1.08**	0.27	0.21	0	98.44
	R6	**0.74**	0.09	0.18	0.01	98.98
Wet	R1	0.03	0.08	**0.26**	0.02	99.61
	R2	0.06	**0.77**	0.42	0.02	98.73
	R3	0.06	**0.78**	0.43	0.03	98.7
	R4	0.07	**0.43**	0.34	0.02	99.14
	R5	0.11	**0.67**	0.57	0.03	98.62
	R6	0.19	0.28	**0.62**	0.06	98.85

*FEAST, fast expectation–maximization microbial source tracking.^*a*^Values in bold indicate that the fecal source was calculated as having the largest relative contribution in total fecal sources.*

To evaluate the accuracy of FEAST for predicting the relative contributions of sources, Spearman’s rank correlations were performed to relate sink predictions with the relative standard deviation (RSD) values obtained from five individual FEAST runs. We observed strong and significant negative correlations between the FEAST predictions and the RSD values (*ρ* = −0.82, *P* < 0.001). Larger relative contributions such as human feces showed lower RSD values (7–16%), whereas smaller source proportions such as sheep had high RSD values (>75%) during the dry season (Supplementary Table 13). Importantly, the source contributing the largest fecal pollution in each sink showed low RSD values (7%–19%; Supplementary Table 13).

## Discussion

In this study, MST methods based on molecular markers and machine learning programs were applied together to distinguish the fecal inputs from multiple sources in a rural river located in Beijing, China. Previous studies have shown the credibility of qPCR assays using markers with high sensitivity and specificity in distinguishing the different fecal sources in the river water (Layton et al., 2013; Zhang et al., 2020). However, the MST method using markers has some limitations, such as detecting only a single specific source of pollution rather than multiple sources at the same time. FEAST, a newly emerging computational tool, could be applied to estimate multiple potential sources and the relative contributions of various fecal inputs at the same time. This study comprehensively combined these two advanced MST methods and found that the relative contributions of fecal pollution in the river were influenced by rainfall events. As such, more measures should be taken to prevent human and animal feces from flowing into rivers during the wet season.

### Evaluation of MST qPCR Assays in Fecal Samples

The universal marker BacUni, the human-associated markers HF183-1 and BacH, swine-associated marker Pig-2-Bac, ruminant-associated Rum-2-Bac, and the avian-associated marker AV4143 performed well for the target and non-target host species in this study. The universal marker BacUni, targeting the 16S rRNA gene of Bacteroidales, was detected in all human and animal fecal samples (Figure 2 and Supplementary Table 8), which was comparable to the 100% sensitivity observed in other regions, including the United States (Kildare et al., 2007), India (Odagiri et al., 2015), Thailand (Somnark et al., 2018), and Kenya (Jenkins et al., 2009). Both HF183-1 and BacH exhibited sensitivity and specificity greater than 91%, whereas the two remaining human-associated markers HF183-2 and BacHum demonstrated specificity less than 80% despite being amplified in all the tested human fecal samples. According to the guideline document of the USEPA (U.S. Environmental Protection Agency, 2005), a marker for MST can be creditable only when its specificity is 80% or higher (maximum value of 100%). Additionally, our results indicated that HF183-1 and BacH had good distinguishing effects, which was consistent with previous studies carried out in China (Vadde et al., 2019; Zhang et al., 2020). The swine-associated marker Pig-2-Bac, the ruminant-associated marker Rum-2-Bac, and the avian-associated marker AV4143 were found to be qPCR-positive in at least 86% of the target host samples tested in this study (Figure 2 and Supplementary Table 8), which was also consistent with other investigations carried out on samples collected from China by He et al. (2016), Liang et al. (2020), Zhang et al. (2020).

The classification method of the 25th/75th metric based on the concentrations of the MST markers can effectively filter out false positives with low concentrations and then improve the reliability of the MST markers. Fecal samples from 13 host species were tested in this study, which met the requirements of the USEPA MST guidelines that more than 10 species of animals should be used for evaluations of host specificity (U.S. Environmental Protection Agency, 2005). Lower false-positive signals from WCRs in the fecal samples could not be detected in the environmental samples because fecal droppings were diluted after entering rivers (Balleste et al., 2020; Zhang et al., 2020). The cross-reactivities of the markers that performed well in sensitivity and specificity (i.e., BacUni, HF183-1, BacH, Pig-2-Bac, Rum-2-Bac, and AV4143) were almost classified as NCRs or WCRs (except an MCR in AV4143 with an equine sample), indicating that the signals from the non-target samples might not be detected in the river water samples. Therefore, the markers BacUni, HF183-1, BacH, Pig-2-Bac, Rum-2-Bac, and AV4143 were considered suitable for subsequent experiments for the detection of the presence or absence of human or animal fecal sources in environmental samples.

### Application of MST Markers in Water Samples

The prevalence of human or animal feces in the Fsq River was related to the presence or absence of rainfall events. The detection rates of the human-associated markers were higher in the samples collected during the dry season than in those collected during the wet season (Table 1), indicating baseline human fecal pollution in the Fsq River. With rainfall flowing into the river, the original concentrations of the human markers in the river water were diluted, resulting in the lower detection rates of human feces, consistent with the results of previous studies (Newton et al., 2013; Balleste et al., 2020). Conversely, the amplified signals of swine feces were only found in the samples from the wet season, but not in the dry season. A similar trend was also observed for Rum-2-Bac, indicating the increased input of ruminant feces owing to runoffs, as previously reported (Sidhu et al., 2012). Therefore, the prevalence difference of the markers between the samples collected during dry and wet weathers demonstrated the influence of bacterial inputs from different fecal sources due to rainfall events. In addition, the detection rates of fecal pollution in the outfall water samples revealed the prevalence rates of human, ruminant, and swine sources from high to low, consistent with the observations in the river water samples, suggesting that outfall water might contribute to pollution of the river.

Human fecal inputs were the most frequently detected in the Fsq River, followed by swine and ruminant fecal inputs, whereas no avian feces were detected. The Fsq River flows through many villages in the studied area, where swine and cattle are bred on a small or a large scale. Outfall water is one of the ways in which rainwater runoffs enter the receiving water. Runoff, especially seasonal rainwater in the wet season, has been widely recognized as a major transport vector of pollutants into the receiving water; therefore, runoff was considered a significant contributor to the deterioration of the quality of rural receiving waters (Barbosa et al., 2012; Kostyla et al., 2015). A higher level of fecal contamination has been observed in research of rural areas compared with that in urban areas during the wet season (Kostyla et al., 2015). Therefore, it is imperative to impose more measures such as farm management or ditch flow control in rural settings during the wet season to improve water quality.

### Application of FEAST Program in Water Samples

Only concentrations of the same marker, rather than different markers, can be used to compare the levels of fecal pollution of the targeting source among different samples. It should be noted that the concentrations of the different markers might not be comparable, especially when these markers were designed from different gene fragments of diverse bacteria, according to previous reports (Liang et al., 2020; Zhang et al., 2020). Therefore, a higher gene copy number might not indicate a higher pollution level of the corresponding host species. In the case of the FEAST program, a “source” library including the potential sources was built, and each sample tested was treated as an individual “sink.” The relative contribution of each source to each sink was calculated according to the matching ratio of the OTUs between the “sink” and the “source” library. Therefore, FEAST could calculate the relative contribution of each source in the tested samples, which cannot be achieved by the MST method based on the qPCR technique. Simultaneous screening of the OTUs of all sources was carried out using the FEAST program to match the OTUs of each sink, and thus the relative contribution of each source in each sink could be identified at the same time.

The construction of the “source” library included local fecal samples from humans, swine, and ruminants (i.e., bovine and sheep) detected in the Fsq River based on the results of the qPCR assays. Firstly, compared with a foreign source library, a local source library could efficiently distinguish fecal sources in the sink samples (Staley et al., 2018), and thus the fecal samples used to build the “source” library were collected from China in this study. Secondly, the prediction results were more reliable when only the known potential sources were included in the “source” library rather than more but random sources (Brown et al., 2019). In addition, highly clustered samples were selected using hierarchical clustering of individual fecal samples to constitute a representative “source” library (Supplementary Figure 1) because the low intragroup variability of the source profiles also enhanced the accuracy of prediction of community-based MST methods (Brown et al., 2019). Analyses of the fecal (*n* = 58) and river water (*n* = 12) samples indicated that the composition of bacterial community differed significantly between the fecal and water samples (Figure 4), ensuring the determination of the presence of fecal inputs and the identification of specific inputs.

fast expectation–maximization microbial source tracking predicted that the largest fecal input in sites R1 to R6 was from human source under dry weather conditions, whereas a bovine or swine source was dominant during the wet season, in agreement with the analysis results using host-associated molecular markers. FEAST is a promising tool to detect low-level bacterial signatures of freshwater, which were similar to those obtained using SourceTracker (O’Dea et al., 2019). FEAST analyses assigned the contamination of the river water samples collected during dry weather to human fecal signatures, comprising 0.34%–3.15% of the total bacterial community (Figure 6 and Table 2). Similar results, in which <10% (Newton et al., 2013) and 1–13% (Ahmed et al., 2015) were assigned to human fecal inputs, were reported in previous studies that investigated the presence of human feces using SourceTracker in water samples. The non-human fecal inputs always constituted <1% of the total proportion of the sink community in the river water samples, even though the contributing percentage of bovine or swine was the highest in the total fecal inputs during wet weather periods. Previous reports have also indicated that non-human fecal inputs were predicted to be trivial when they were compared with environmental samples near the sampling regions (Newton et al., 2013; Baral et al., 2018).

The results using the FEAST program validated its use as a tool to distinguish fecal pollution of various sources and indicated that it could be a promising addition to the toolbox for identifying pollution sources and for suggesting appropriate migration strategies to improve water quality. Of course, this study also had some limitations, even though the predicted results of the FEAST program were promising. For example, only a very small sample size was analyzed by the FEAST program and only fecal samples were included to build the “source” library. In this study, most microbial taxa in the sink did not match the fecal signature in the “source” library, thus being classified as unknown, as in the case of published studies using SourceTracker (Newton et al., 2013; Staley et al., 2018; O’Dea et al., 2019). Therefore, more samples, especially potential sources near the sampling sites (e.g., soil and rainwater samples), need to be included in the “source” library if the composition of the unknown source is to be clarified. SourceTracker, a community-based program like FEAST, could accurately predict the fecal compositions of fecal sources, according to a previous double-blinded study (Staley et al., 2018). In previous studies using SourceTracker, unknown sources were mainly identified as treated effluent (Henry et al., 2016), wastewater effluent or influent (Ahmed et al., 2017; Brown et al., 2017), and embankment soil or streambed sediment (Baral et al., 2018; Li et al., 2020), which were collected in the studied area near the sink samples.

## Conclusion

The results of the FEAST program were consistent with those of the qPCR assays. Both methods revealed that fecal contamination from humans was dominant during dry weather and that the baseline of human fecal pollution might exist in the Fsq River. Swine and ruminant fecal sources were more prevalent in the samples during the wet season than in those during the dry season, owing to their potential discharge into the river water *via* the runoff system. MST methods using molecular markers retain certain advantages because they can monitor the sources of pollutants quickly and accurately without building the “source” library, especially when the sample size is small. However, there is no doubt that FEAST based on machine learning could provide promising advantages over traditional culture-based fecal indicator approaches and single-target qPCR assays. The method only needs to characterize relevant source samples instead of building thousands of libraries, thus reducing the costs or labor. Of course, each MST approach has its limitations, and the FEAST program is no exception. The development of a representative library requires time, but efficient and rapid advantages of the method could be fully exploited in the long-term dynamic monitoring of environmental samples from multiple pollution sources once a representative library is created. Therefore, the future development of FEAST should not only focus on its technical limitations but should also make better use of the technology academically and practically so that it could be better applied to different avenues of microbial community research, such as identifying contamination, environment mixing, or microbial migration.

## Data Availability Statement

The datasets presented in this study can be found in online repositories. The names of the repository/repositories and accession number(s) can be found below: SRA database (NCBI) under the BioProject accession number PRJNA713417, with Biosample accession numbers SAMN18274560–SAMN18274616 and SAMN18252387–SAMN18252398. Besides, sequence data of one bovine feces has been deposited in the SRA database (NCBI) under the BioProject accession number PRJNA392724, with biosample number SAMN07305415.

## Author Contributions

ZSY and HXL designed the experiment. HXL and BBW carried out the field sampling. HXL, BBW, and FN performed the laboratory work. HXL and ZSY analyzed the data and wrote the manuscript. RYL, HXZ, and GW contributed to the revision of the manuscript. All authors contributed to the article and approved the submitted version.

## Conflict of Interest

The authors declare that the research was conducted in the absence of any commercial or financial relationships that could be construed as a potential conflict of interest.
