# Supplementary material for: Synergistic Application of Molecular Markers and Community-Based Microbial Source Tracking Methods for Identification of Fecal Pollution in River Water During Dry and Wet Seasons
Source: Front Microbiol. 2021 Jun 14;12:660368. doi: 10.3389/fmicb.2021.660368 (PMC8236858; doi:10.3389/fmicb.2021.660368)
Supplement: Supplementary file 1 [file Data_Sheet_1.docx]

**
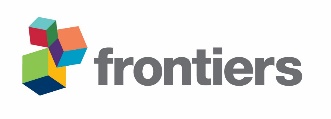
**

Supplementary Material

Number of supplementary tables: 13

Number of supplementary figures: 2

**TABLE S1**. Information on fecal samples collected from human and animals

| Fecal source | Scientific name | Number tested | Number for qPCR | Number for FEAST (Source) |
| --- | --- | --- | --- | --- |
| Human | *Homo sapiens* | 28 | 13 | 21 |
| Swine | *Sus domesticus* | 20 | 10 | 16 |
| Canine | *Canis lupus familiaris* | 6 | 6 | / |
| Equine | *Equus ferus caballus* | 11 | 6 | / |
| Donkey | *Equus africanus asinus* | 4 | 4 | / |
| Bovine | *Bos taurus domestica* | 14 | 6 | 8 |
| Sheep | *Ovis aries* | 18 | 5 | 13 |
| Goat | *Capra aegagrus hircus* | 5 | 5 | / |
| Chicken | *Gallus gallus domesticus* | 12 | 12 | / |
| Duck | *Cairna moschata* | 10 | 10 | / |
| Goose | *Cygnus cygnus* | 10 | 10 | / |
| Pigeon | *Columba livia domestica* | 9 | 9 | / |
| Fish | *Ctenopharyngodon* | 37 | 37 | / |
| **Total** |  | **184** | **133** | **58** |

**TABLE S2.** Information about sampling locations of river and outfall water along with coordinates

| Samples | No. of samples | Number of samples | Sampling events |
| --- | --- | --- | --- |
| River water | R1 | 5 | BF,AF1,AF2,AF3,AF4 |
| (n=80) | R2 | 5 | BF,AF1,AF2,AF3,AF4 |
|  | R3 | 5 | BF,AF1,AF2,AF3,AF4 |
|  | R4 | 4 | BF, AF2,AF3,AF4 |
|  | R5 | 5 | BF,AF1,AF2,AF3,AF4 |
|  | R6 | 5 | BF,AF1,AF2,AF3,AF4 |
|  | R7 | 5 | BF,AF1,AF2,AF3,AF4 |
|  | R8 | 5 | BF,AF1,AF2,AF3,AF4 |
|  | R9 | 3 | AF1, AF3,AF4 |
|  | R10 | 5 | BF,AF1,AF2,AF3,AF4 |
|  | R11 | 5 | BF,AF1,AF2,AF3,AF4 |
|  | R12 | 5 | BF,AF1,AF2,AF3,AF4 |
|  | R13 | 5 | BF,AF1,AF2,AF3,AF4 |
|  | R14 | 5 | BF,AF1,AF2,AF3,AF4 |
|  | R15 | 5 | BF,AF1,AF2,AF3,AF4 |
|  | R16 | 3 | AF2,AF3,AF4 |
|  | R17 | 5 | BF,AF1,AF2,AF3,AF4 |
| Outfall water | FR2 | 4 | AF1,AF2,AF3,AF4 |
| (n=16) | FR7 | 5 | BF,AF1,AF2,AF3,AF4 |
|  | FR9 | 3 | AF2,AF3,AF4 |
|  | FR14 | 2 | AF2,AF3 |
|  | FR15 | 2 | AF2,AF3 |
| **Total** | **96** |  |  |

**Table S3.** Information of oligonucleotide primers and probes of MST markers using TaqMan real-time quantitative PCR

| Assays | Primer/probe | Sequence（5’-3’） | Concentration（nM） | Annealing temperature (℃) | Size of product（bp） |
| --- | --- | --- | --- | --- | --- |
| **Universal** |  |  |  |  |  |
| BacUni (Kildare et al., 2007) | BacUni-520f | CGTTATCCGGATTTATTGGGTTTA | 400 | 60 | 170 |
|  | BacUni-690r1 | CAATCGGAGTTCTTCGTGATATCTA | 400 |  |  |
|  | BacUni-690r2 | AATCGGAGTTCCTCGTGATATCTA | 400 |  |  |
|  | BacUni-656p | 6-FAM^a^-TGGTGTAGCGGTGAAA-MGB^b^ | 80 |  |  |
| **Human** |  |  |  |  |  |
| HF183-1 | HF183F (Bernhard et al., 2000) | ATCATGAGTTCACATGTCCG | 1000 | 60 | 126 |
|  | BacR287 (Green et al., 2014) | CTTCCTCTCAGAACCCCTATCC | 1000 |  |  |
|  | BacP234 (Green et al., 2014) | FAM^c^-CTAATGGAACGCATCCC-MGB | 80 |  |  |
| BacH (Reischer et al., 2007) | BacHF | CTTGGCCAGCCTTCTGAAAG | 200 | 60 | 93 |
|  | BacHR | CCCCATCGTCTACCGAAAATAC | 200 |  |  |
|  | BacH-pC | FAM-TCATGATCCCATCCTG-MGB | 100 |  |  |
|  | BacH_pT | FAM-TCATGATGCCATCTTG-MGB | 100 |  |  |
| HF183-2 | HF183F (Bernhard et al., 2000) | ATCATGAGTTCACATGTCCG | 1000 | 60 | 167 |
|  | BFDRev (Converse et al., 2009) | CGTAGGAGTTTGGACCGTGT | 1000 |  |  |
|  | BFDFam(Converse et al., 2009) | FAM-CTGAGAGGAAGGTCCCCCACATTGGA-TAMRA^d^ | 80 |  |  |
| BacHum (Kildare et al., 2007) | BacHum-241R | CGTTACCCCGCCTACTATCTAATG | 400 | 60 | 81 |
|  | BacHum-160F | TGAGTTCACATGTCCGCATGA | 400 |  |  |
|  | BacHum-193p | 6-FAM-TCCGGTAGACGATGGGGATGCGTT-TAMRA | 80 |  |  |
| **Swine** |  |  |  |  |  |
| Pig-2-Bac (Mieszkin et al., 2009) | Pig-2-Bac41F | GCATGAATTTAGCTTGCTAAATTTGAT | 300 | 60 | 116 |
|  | Pig-2-Bac163R | ACCTCATACGGTATTAATCCGC | 300 |  |  |
|  | Pig-2-Bac113P | VIC^e^-TCCACGGGATAGCC-MGB | 200 |  |  |
| **Ruminant** |  |  |  |  |  |
| Rum-2-Bac (Mieszkin et al., 2010) | BacB2-590F | ACAGCCCGCGATTGATACTGGTAA | 200 | 60 | 99 |
|  | Bac708Rm | CAATCGGAGTTCTTCGTGAT | 200 |  |  |
|  | BacB2-626P | FAM-ATGAGGTGGATGGAATTCGTGGTGT-BHQ-1^f^ | 200 |  |  |
| **Avian** |  |  |  |  |  |
| AV4143 | AV4143F (Ohad et al., 2016) | TGCAAGTCGAACGAGGATTTCT | 500 | 60 | 244 |
|  | AV4143R (Ohad et al., 2016) | TCACCTTGGTAGGCCGTTACC | 500 |  |  |
|  | AV4143P1 (Liang et al., 2020) | 6-FAM-AGATGGTTCTGCTATCGCTTT-BHQ1 | 250 |  |  |

^a^ 6-FAM: 6- Carboxy-fluorescein

^b^ MGB: Minor Groove Binder

^c^ FAM: Carboxy-fluorescein

^d^ TAMRA: Carboxytetramethylrhodamine

^e^ VIC: 2′-chloro-7′-phenyl-1,4-dichloro-6-carboxy fluorescein

^f^ BHQ1: Black Hole Quencher 1

**TABLE S4.** Sample format under “FEAST example Multiple sinks” script

| Sample ID | Env | SourceSink | id |
| --- | --- | --- | --- |
| R1_BF | Water1 | Sink | 1 |
| R2_BF | Water2 | Sink | 2 |
| R3_BF | Water3 | Sink | 3 |
| R4_BF | Water4 | Sink | 4 |
| R5_BF | Water5 | Sink | 5 |
| R6_BF | Water6 | Sink | 6 |
| R1_AF3 | Water7 | Sink | 7 |
| R2_AF3 | Water8 | Sink | 8 |
| R3_AF3 | Water9 | Sink | 9 |
| R4_AF3 | Water10 | Sink | 10 |
| R5_AF3 | Water11 | Sink | 11 |
| R6_AF3 | Water12 | Sink | 12 |
| HU1 | Human1 | Source | NA |
| HU2 | Human2 | Source | NA |
| HU3 | Human3 | Source | NA |
| HU4 | Human4 | Source | NA |
| HU5 | Human5 | Source | NA |
| HU6 | Human6 | Source | NA |
| HU7 | Human7 | Source | NA |
| HU8 | Human8 | Source | NA |
| HU9 | Human9 | Source | NA |
| HU10 | Human10 | Source | NA |
| HU11 | Human11 | Source | NA |
| HU12 | Human12 | Source | NA |
| HU13 | Human13 | Source | NA |
| HU14 | Human14 | Source | NA |
| HU15 | Human15 | Source | NA |
| HU16 | Human16 | Source | NA |
| HU17 | Human17 | Source | NA |
| HU18 | Human18 | Source | NA |
| HU19 | Human19 | Source | NA |
| HU20 | Human20 | Source | NA |
| HU21 | Human21 | Source | NA |
| SW1 | Swine1 | Source | NA |
| SW2 | Swine2 | Source | NA |
| SW3 | Swine3 | Source | NA |
| SW4 | Swine4 | Source | NA |
| SW5 | Swine5 | Source | NA |
| SW6 | Swine6 | Source | NA |
| SW7 | Swine7 | Source | NA |
| SW8 | Swine8 | Source | NA |
| SW9 | Swine9 | Source | NA |
| SW10 | Swine10 | Source | NA |
| SW11 | Swine11 | Source | NA |
| SW12 | Swine12 | Source | NA |
| SW13 | Swine13 | Source | NA |
| SW14 | Swine14 | Source | NA |
| SW15 | Swine15 | Source | NA |
| SW16 | Swine16 | Source | NA |
| CO1 | Bovine1 | Source | NA |
| CO2 | Bovine2 | Source | NA |
| CO3 | Bovine3 | Source | NA |
| CO4 | Bovine4 | Source | NA |
| CO5 | Bovine5 | Source | NA |
| CO6 | Bovine6 | Source | NA |
| CO7 | Bovine7 | Source | NA |
| CO8 | Bovine8 | Source | NA |
| SP1 | Sheep1 | Source | NA |
| SP2 | Sheep2 | Source | NA |
| SP3 | Sheep3 | Source | NA |
| SP4 | Sheep4 | Source | NA |
| SP5 | Sheep5 | Source | NA |
| SP6 | Sheep6 | Source | NA |
| SP7 | Sheep7 | Source | NA |
| SP8 | Sheep8 | Source | NA |
| SP9 | Sheep9 | Source | NA |
| SP10 | Sheep10 | Source | NA |
| SP11 | Sheep11 | Source | NA |
| SP12 | Sheep12 | Source | NA |
| SP13 | Sheep13 | Source | NA |

**TABLE S5**. BLAST information about target sequences contained in standards for qPCR assays

| Host | qPCR assays | Size of PCR product/bp | Per. Identity | Query cover | Accession | Description |
| --- | --- | --- | --- | --- | --- | --- |
| Total | BacUni | 177 | 99.43% | 100% | NR_041277.1 | *Bacteroides* spp. |
| Human | HF183-1 | 124 | 100% | 98.41% | FJ219782.1 | *Bacteroides* sp. |
|  | BacH | 93 | 98.94% | 100% | MT464394.1 | *Bacteroides dorei* |
|  | HF183-2 | 167 | 100% | 99% | MT464394.1 | *Bacteroides dorei* |
|  | BacHum | 82 | 100% | 100% | MT464394.1 | *Bacteroides dorei* |
| Swine | Pig-2-Bac | 117 | 100% | 100% | HQ201809.1 | *Bacteroidales* |
| Ruminant | Rum-2-Bac | 100 | 100% | 100% | HM100675.1 | *Bacteroidales* |
| Avian | AV4143 | 244 | 100% | 98.77% | MT597565.1 | *Lactobacillus salivarius* |

**TABLE S6.** Performance characteristics for all qPCR assays

| Assay | Slope | Y-intercept | R^2^ | Efficiency/% | LOD^a^  (copies/reaction) | LOQ^b^  (copies/reaction) |
| --- | --- | --- | --- | --- | --- | --- |
| BacUni | -3.341 | 39.771 | 0.999 | 99.2 | 5 | 200 |
| HF183F-1 | -3.369 | 40.448 | 1 | 98.1 | 70 | 2000 |
| HF183F-2 | -3.275 | 39.713 | 1 | 101.9 | 50 | 500 |
| BacHum | -3.322 | 40.466 | 0.999 | 99.9 | 50 | 400 |
| BacH | -3.581 | 43.138 | 0.999 | 90.2 | 300 | 2000 |
| Pig-2-Bac | -3.675 | 42.443 | 1 | 87.1 | 100 | 500 |
| Rum-2-Bac | -3.382 | 40.465 | 0.999 | 99.8 | 200 | 1000 |
| AV4143 | -3.315 | 38.899 | 0.999 | 100.3 | 25 | 40 |

^a^ Limit of Detection

^b^ Limit of Quantification

**TABLE S7.** Reproducibility tested by standards (103 to 108 copies/μL of the positive control) of each marker on different reaction plates

|  | Mean Ct | SD^a^ | CV^b^ |  | Mean Ct | SD^a^ | CV^b^ |  | Mean Ct | SD^a^ | CV^b^ |  | Mean Ct | SD^a^ | CV^b^ |  | Mean Ct | SD^a^ | CV^b^ |  | Mean Ct | SD^a^ | CV^b^ |
| --- | --- | --- | --- | --- | --- | --- | --- | --- | --- | --- | --- | --- | --- | --- | --- | --- | --- | --- | --- | --- | --- | --- | --- |
|  | Ct values of 10^8^ copies/µl | | |  | Ct values of 10^7^ copies/µl | | |  | Ct values of 10^6^ copies/µl | | |  | Ct values of 10^5^copies/µl | | |  | Ct values of 10^4^ copies/µl | | |  | Ct values of 10^3^ copies/µl | | |
| BacUni | 13.9 | 0.4 | 3.0% |  | 17.5 | 0.5 | 3.0% |  | 21.3 | 0.5 | 3.0% |  | 24.9 | 0.8 | 3.0% |  | 28.3 | 0.6 | 2.0% |  | 32.1 | 0.8 | 2.0% |
| HF183-1 | 11.5 | 0.4 | 3.0% |  | 15.0 | 0.5 | 3.0% |  | 18.4 | 0.5 | 3.0% |  | 22.0 | 0.6 | 3.0% |  | 25.8 | 0.8 | 3.0% |  | 29.3 | 0.9 | 3.0% |
| BacH | 11.7 | 0.4 | 4.0% |  | 15.3 | 0.4 | 3.0% |  | 19.0 | 0.3 | 2.0% |  | 22.7 | 0.5 | 2.0% |  | 26.5 | 0.6 | 2.0% |  | 30.4 | 0.6 | 2.0% |
| HF183-2 | 11.7 | 0.4 | 3.0% |  | 15.4 | 0.5 | 3.0% |  | 19.1 | 0.6 | 3.0% |  | 22.7 | 0.7 | 3.0% |  | 26.4 | 0.9 | 3.0% |  | 29.9 | 0.9 | 3.0% |
| BacHum | 12.0 | 0.7 | 6.0% |  | 15.6 | 0.9 | 6.0% |  | 19.2 | 1.1 | 6.0% |  | 22.8 | 1.2 | 5.0% |  | 26.3 | 1.4 | 5.0% |  | 29.7 | 1.4 | 5.0% |
| Pig-2-Bac | 9.5 | 0.5 | 5.0% |  | 13.2 | 0.6 | 5.0% |  | 16.9 | 0.7 | 4.0% |  | 20.6 | 0.7 | 3.0% |  | 24.5 | 0.5 | 2.0% |  | 28.1 | 0.6 | 2.0% |
| Rum-2-Bac | 12.1 | 0.6 | 5.0% |  | 15.5 | 0.7 | 5.0% |  | 19.0 | 0.9 | 5.0% |  | 22.5 | 1.0 | 5.0% |  | 25.9 | 1.2 | 5.0% |  | 29.5 | 1.3 | 4.0% |
| AV4143 | 9.9 | 0.5 | 5.0% |  | 12.9 | 0.5 | 4.0% |  | 16.7 | 0.9 | 5.0% |  | 20.2 | 0.8 | 4.0% |  | 24.2 | 1.0 | 4.0% |  | 27.9 | 1.0 | 3.0% |
| Mean ± SD^a^ of CV^b^ | (4.0 ± 0.1)% | | |  | (4.0 ± 0.1)% | | |  | (4.0 ± 0.1)% | | |  | (3.0 ± 0.1)% | | |  | (3.0 ± 0.1)% | | |  | (3.0 ± 0.1)% | | |

^a^ Standard deviation

^b^ Percentage of the coefficient of variation

**TABLE S8.** Qualitative and quantitative analyses of MST markers in fecal samples from human and animal tested

| Sources | No. of samples | Total | Human |  |  |  | Swine | Ruminant | Avian |
| --- | --- | --- | --- | --- | --- | --- | --- | --- | --- |
|  |  | BacUni | HF183-1 | BacH | HF183-2 | BacHum | Pig-2-Bac | Rum-2-Bac | AV4143^a^ |
|  |  | Mean ± SD^b (n = number of samples within LOD)^ | Mean ± SD^b (n = number of samples within LOD)^ | Mean ± SD^b (n = number of samples within LOD)^ | Mean ± SD^b (n = number of samples within LOD)^ | Mean ± SD^b (n = number of samples within LOD)^ | Mean ± SD^b (n = number of samples within LOD)^ | Mean ± SD^b (n = number of samples within LOD)^ | Mean ± SD^b (n = number of samples within LOD)^ |
| Human | 13 | 9.01±0.23^(n=13)^ | 6.16±1.48^(n=13)^ | 6.51±1.01^(n=13)^ | 6.20±1.21 ^(n=13)^ | 6.22±0.95 ^(n=13)^ | 2.47±0.19^(n=3)^ | 0^(n=0)^ | 0^(n=0/13)^ |
| Swine | 10 | 8.41±0.36^(n=10)^ | 0^(n=0)^ | 0^(n=0)^ | 3.20±0.39^(n=6)^ | 3.52^(n=1)^ | 6.84±0.84^(n=10)^ | 0^(n=0)^ | 3.09 ^(n=1/10)^ |
| Canine | 6 | 7.25±1.07^(n=6)^ | 3.40±0.02^(n=2)^ | 4.58±0.55^(n=3)^ | 2.48^(n=1)^ | 2.90±0.01^(n=2)^ | 0^(n=0)^ | 0^(n=0)^ | 2.43 ^(n=1/6)^ |
| Equine | 6 | 7.41±0.21^(n=6)^ | 3.27 ^(n=1)^ | 0 ^(n=0)^ | 3.80±0.02 ^(n=2)^ | 3.12(n=1) | 0^(n=0)^ | 3.45^(n=1)^ | 3.68 ^(n=1/6)^ |
| Donkey | 4 | 8.00±0.24^(n=4)^ | 0^(n=0)^ | 0^(n=0)^ | 3.46±0.59^(n=2)^ | 3.55±0.03^(n=4)^ | 0^(n=0)^ | 0^(n=0)^ | 0 ^(n=0/5)^ |
| Bovine | 6 | 8.54±0.23^(n=6)^ | 3.38±0.15^(n=2)^ | 0^(n=0)^ | 3.53±0.75^(n=6)^ | 3.58±0.04^(n=6)^ | 0^(n=0)^ | 6.92±0.24^(n=6)^ | 0 ^(n=0/6)^ |
| Sheep | 5 | 8.81±0.20^(n=5)^ | 3.48^(n=1)^ | 0(^n=0)^ | 2.53±0.06^(n=2)^ | 3.77±0.40^(n=4)^ | 0^(n=0)^ | 6.91±0.67^(n=5)^ | 0 ^(n=0/5)^ |
| Goat | 5 | 8.67±0.35^(n=5)^ | 0^(n=0)^ | 0(^n=0)^ | 2.62^(n=1)^ | 0(^n=0)^ | 3.23^(n=1)^ | 7.24±0.55^(n=5)^ | 0 ^(n=0/5)^ |
| Chicken | 12 | 5.82±1.64^(n=12)^ | 0^(n=0)^ | 0^(n=0)^ | 0^(n=0)^ | 3.50^(n=1)^ | 0^(n=0)^ | 0^(n=0)^ | 4.07±0.94 ^(n=75/75)^ |
| Duck | 10 | 5.69±1.24^(n=10)^ | 3.96±0.58^(n=2)^ | 2.91±0.52^(n=3)^ | 3.42±0.63^(n=3)^ | 3.88±0.53^(n=2)^ | 0^(n=0)^ | 0^(n=0)^ | 2.25±0.04 ^(n=2/10)^ |
| Goose | 10 | 5.32±1.53^(n=10)^ | 0^(n=0)^ | 0^(n=0)^ | 0^(n=0)^ | 0(^n=0)^ | 0^(n=0)^ | 3.37^(n=1)^ | 2.18±0.71 ^(n=7/14)^ |
| Pigeon | 9 | 3.99±0.56^(n=9)^ | 0^(n=0)^ | 2.93±0.48^(n=2)^ | 2.52±0.61^(n=3)^ | 3.59^(n=1)^ | 0^(n=0)^ | 0^(n=0)^ | 2.76±0.78 ^(n=5/6)^ |
| Fish | 37 | 5.81±0.39^(n=37)^ | 3.20±0.27^(n=2)^ | 2.78±0.20^(n=3)^ | 3.18±0.03^(n=3)^ | 3.73±0.34^(n=6)^ | 0^(n=0)^ | 0^(n=0)^ | 0 ^(n=0/37)^ |
| Qualitative analysis | Sensitivity (%) | 100 | 100 | 100 | 100 | 100 | 100 | 100 | 86 |
|  | Specificity (%) | / | 93 | 91 | 76 | 77 | 97 | 98 | 97 |
|  | Accuracy (%) | / | 93 | 92 | 78 | 79 | 97 | 98 | 91 |
| Quantitative analysis | Target | 6.70±1.71^(n=133)^ | 6.16±1.48^(n=13)^ | 6.51±1.01^(n=13)^ | 6.20±1.21 ^(n=13)^ | 6.22±0.95 ^(n=13)^ | 6.84±0.84^(n=10)^ | 7.02±0.50^(n=16)^ | 3.81±1.09^(n=92)^ |
|  | Non-target | NA | 3.46±0.35^(n=10)^ | 3.33±0.89^(n=11)^ | 3.19±0.61^(n=29)^ | 3.59±0.33^(n=28)^ | 2.66±0.41^(n=14)^ | 3.41±0.06^(n=2)^ | 3.1±0.6^(n=3)^ |

^a^ Sample numbers of marker AV4143 in fecal samples from human and fish were tested in this study, and that of from other animal was determined in the previous study (Liang et al., 2020).

^b^ Standard deviation.

**TABLE S9.** Classification of cross-reactivity for candidate host-associated markers

| **HF183/1** | | Human | Swine | Canine | Equine | Donkey | Bovine | Sheep | Goat | Chicken | Duck | Goose | Pigeon | Fish |
| --- | --- | --- | --- | --- | --- | --- | --- | --- | --- | --- | --- | --- | --- | --- |
| Target | 25th ( Log10 GC/g ) | 4.16 | / | / | / | / | / | / | / | / | / | / | / | / |
| Non-target | 75th ( Log10 GC/g) | / | / | 3.40 | 3.27 | / | 3.44 | 3.48 | / | / | 4.16 | / | / | 3.30 |
| 25th/75th metric^a^ | | / |  | 2.76 | 2.89 | / | 2.72 | 2.68 | / | / | 2.00 | / | / | 2.86 |
| HF183-1 Classification^b^ | | TP | NCR | WCR | WCR | NCR | NCR | NCR | NCR | NCR | WCR | NCR | NCR | WCR |
| **BacH** | | Human | Swine | Canine | Equine | Donkey | Bovine | Sheep | Goat | Chicken | Duck | Goose | Pigeon | Fish |
| Target | 25th ( Log10 GC/g ) | 5.20 | / | / | / | / | / | / | / | / | / | / | / | / |
| Non-target | 75th ( Log10 GC/g) | / | / | 4.82 | / | / | / | / | / | / | 3.15 | / | 3.10 | 2.89 |
| 25th/75th metric^a^ | | / | / | 0.38 | / | / | / | / | / | / | 2.05 | / | 2.10 | 2.31 |
| BacH Classification^b^ | | TP | NCR | WCR | NCR | NCR | NCR | NCR | NCR | NCR | WCR | NCR | WCR | WCR |
| **Pig-2-Bac** | | Human | Swine | Canine | Equine | Donkey | Bovine | Sheep | Goat | Chicken | Duck | Goose | Pigeon | Fish |
| Target | 25th ( Log10 GC/g ) | / | 6.55 | / | / | / | / | / | / | / | / | / | / | / |
| Non-target | 75th ( Log10 GC/g) | 2.53 | / | / | / | / | / | / | / | / | / | / | / | / |
| 25th/75th metric^a^ | |  | / | / | / | / | / | / | / | / | / | / | / | / |
| Pig-2-Bac Classification^b^ | | WCR | TP | NCR | NCR | NCR | NCR | NCR | NCR | NCR | NCR | NCR | NCR | NCR |
| **Rum-2-Bac** | | Human | Swine | Canine | Equine | Donkey | Bovine | Sheep | Goat | Chicken | Duck | Goose | Pigeon | Fish |
| Target | 25th ( Log10 GC/g ) | / | / | / | / | / | 6.77 |  |  | / | / | / | / | / |
| Non-target | 75th ( Log10 GC/g) | / | / | / | 3.45 | / | / | / | / | / | / | 3.37 | / | / |
| 25th/75th metric^a^ | | / | / | / | 3.32 | / | / | / | / | / | / | 3.40 | / | / |
| Rum-2-Bac Classification^b^ | | NCR | NCR | NCR | WCR | NCR | TP | TP | TP | NCR | NCR | WCR | NCR | NCR |
| **AV4143** | | Human | Swine | Canine | Equine | Donkey | Bovine | Sheep | Goat | Chicken | Duck | Goose | Pigeon | Fish |
| Target | 25th ( Log10 GC/g ) | / | / | / | / | / | / | / | / | 3.12 |  |  | / | / |
|  | Mean ( Log10 GC/g ) |  |  |  |  |  |  |  |  | 3.76 |  |  |  |  |
| Non-target | 75th ( Log10 GC/g) | / | 3.09 | 2.43 | 3.68 | / | / | / | / | / | / | / | / | / |
|  | Mean ( Log10 GC/g ) |  |  |  | 3.68 |  |  |  |  |  |  |  |  |  |
| 25th/75th metric^a^ | | / | 0.03 | 0.69 | -0.56 | / | / | / | / | / | / | / | / | / |
| AV4143 Classification^b^ | | NCR | WCR | WCR | MCR | NCR | NCR | NCR | NCR | TP | TP | TP | TP | NCR |

^a^ 25th/75th metric = 25th percentile_target_ − 75th percentile_non-target_ (Reischer et al., 2013)

^b^ NCR (no cross-reactivity), the marker did not show any positive signals in the non-target samples; WCR (weak cross-reactivity), the “25th/75th metric” > 0; MCR (moderate cross-reactivity), the “25th/75th metric” < 0; SCR (strong cross-reactivity), the disparity between mean gene copies of target and non-target samples was below 1 order of magnitude or mean gene copy of non-target samples is higher than that in target samples (Zhang et al., 2020).

**TABLE S10.** Qualitative and quantitative analyses of MST markers in tested river water and outfall water samples

| Source | Season | Sampling events | No. of samples | Total | Human |  | Swine | Ruminant | Avian |
| --- | --- | --- | --- | --- | --- | --- | --- | --- | --- |
|  |  |  |  | BacUni | HF183-1 | BacH | Pig-2-Bac | Rum-2-Bac | AV4143 |
|  |  |  |  | Mean ± SD^(n = number of samples within LOD)^ | Mean ± SD^(n = number of samples within LOD)^ | Mean ± SD^(n = number of samples within LOD)^ | Mean ± SD^(n = number of samples within LOD)^ | Mean ± SD^(n = number of samples within LOD)^ | Mean ± SD^(n = number of samples within LOD)^ |
| River water | Dry | BF | 15 | 6.53±0.84^(n=15)^ | 4.34±0.82^(n=13)^ | 4.25±0.90^(n=14)^ | 0^(n=0)^ | 1.54^(n=1)^ | 0^(n=0)^ |
|  | Wet | AF | 65 | 6.13±0.60^(n=65)^ | 3.55±0.56^(n=35)^ | 3.40±0.56^(n=41)^ | 2.50±0.05^(n=2)^ | 2.97±0.65^(n=22)^ | 0^(n=0)^ |
|  |  |  | AF1=16 | 6.58±0.53^(n=16)^ | 4.04±0.45^(n=12)^ | 3.76±0.58^(n=12)^ | 2.53^(n=1)^ | 2.68±0.59^(n=6)^ | 0^(n=0)^ |
|  |  |  | AF2=15 | 6.25±0.43^(n=15)^ | 3.26±0.46^(n=11)^ | 3.35±0.49^(n=13)^ | 0^(n=0)^ | 3.11±0.55^(n=11)^ | 0^(n=0)^ |
|  |  |  | AF3=17 | 5.99±0.47^(n=17)^ | 3.34±0.35^(n=5)^ | 3.20±0.51^(n=7)^ | 0^(n=0)^ | 3.29±0.60^(n=5)^ | 0^(n=0)^ |
|  |  |  | AF4=17 | 5.71±0.59^(n=17)^ | 3.27±0.43^(n=7)^ | 3.10±0.43^(n=9)^ | 2.47^(n=1)^ | 0^(n=0)^ | 0^(n=0)^ |
|  | **Total** |  | **80** | **6.20±0.66^(n=80)^** | **3.76±0.72 ^(n=48)^** | **3.61±0.75^(n=55)^** | **2.50±0.05^(n=2)^** | **2.97±0.65^(n=23)^** | **0^(n=0)^** |
| Outfall water | Dry | BF | 1 | 6.42^(n=1)^ | 3.47^(n=1)^ | 3.50^(n=1)^ | 0^(n=0)^ | 0^(n=0)^ | 0^(n=0)^ |
|  | Wet | AF | 15 | 6.15±1.03^(n=15)^ | 4.06±0.91^(n=5)^ | 3.46±1.25^(n=8)^ | 2.47^(n=1)^ | 2.93±1.16^(n=2)^ | 0^(n=0)^ |
|  |  |  | AF1=2 | 6.26±0.33^(n=2)^ | 3.45^(n=1)^ | 3.23±0.89^(n=2)^ | 0^(n=0)^ | 0^(n=0)^ | 0^(n=0)^ |
|  |  |  | AF2=5 | 6.42±1.13^(n=5)^ | 4.12±0.89^(n=3)^ | 3.55±1.31^(n=4)^ | 2.47^(n=1)^ | 0^(n=0)^ | 0^(n=0)^ |
|  |  |  | AF3=5 | 6.14±1.26^(n=5)^ | 4.92^(n=1)^ | 5.44^(n=1)^ | 0^(n=0)^ | 2.93±1.16^(n=2)^ | 0^(n=0)^ |
|  |  |  | AF4=3 | 5.01±0.40^(n=3)^ | 0^(n=0)^ | 2.89^(n=1)^ | 0^(n=0)^ | 0^(n=0)^ | 0^(n=0)^ |
|  | **Total** |  | **16** | **6.07±1.05^(n=16)^** | **4.03±0.78^(n=6)^** | **3.56±1.16^(n=9)^** | **2.47^(n=1)^** | **2.93±1.16^(n=2)^** | **0 ^(n=0)^** |

**TABLE S11.** Diversity indices of all water and fecal samples for high-throughput sequencing in this study

| Samples | Group | Number of samples | Number of OTUs | Shannon Index | Average sequences |
| --- | --- | --- | --- | --- | --- |
| Water (Sink) | Dry season | 6 | 2897 | 3.81 ± 0.15 | 47767 |
|  | Wet season | 6 | 4809 | 6.03 ± 0.24 | 48809 |
|  | Total | 12 | 5561 | 4.92 ± 1.18 | 48288 |
| Feces (Source) | Human | 21 | 817 | 3.50 ± 0.43 | 40334 |
|  | Swine | 16 | 1979 | 4.03 ± 0.71 | 45275 |
|  | Bovine | 8 | 2080 | 5.16 ± 0.32 | 27455 |
|  | Sheep | 13 | 2184 | 5.39 ± 0.33 | 25159 |
|  | Total | 58 | 3666 | 4.30 ± 0.93 | 36515 |

**TABLE S12.** The average relative abundance of the bacterial community on order level

| Order | Fecal samples | | | | River water samples | |
| --- | --- | --- | --- | --- | --- | --- |
|  | Human | Swine | Bovine | Sheep | Dry season | Wet season |
| *Bacteroidales* | 25.92% | 39.12% | 30.60% | 35.43% | 1.44% | 0.54% |
| *Oscillospirales* | 14.88% | 12.23% | 30.72% | 29.24% | 0.08% | 0.03% |
| *Lachnospirales* | 28.71% | 5.42% | 7.10% | 7.94% | 0.16% | 0.08% |
| *Flavobacteriales* | 0.00% | 0.00% | 0.07% | 0.03% | 41.06% | 5.53% |
| *Burkholderiales* | 0.96% | 0.33% | 0.28% | 0.01% | 27.34% | 15.74% |
| *Peptostreptococcales-Tissierellales* | 2.10% | 4.04% | 8.83% | 2.48% | 0.09% | 0.78% |
| *Christensenellales* | 0.37% | 3.53% | 3.00% | 7.53% | 0.01% | 0.05% |
| *Lactobacillales* | 1.02% | 11.73% | 0.05% | 0.02% | 0.35% | 0.10% |
| *Veillonellales-Selenomonadales* | 2.64% | 8.31% | 0.07% | 0.04% | 0.33% | 0.00% |
| *Clostridiales* | 2.24% | 5.77% | 1.38% | 0.44% | 0.20% | 0.82% |
| *Enterobacterales* | 7.79% | 0.12% | 0.05% | 0.02% | 0.75% | 0.09% |
| *Micrococcales* | 0.01% | 0.00% | 0.02% | 0.02% | 6.40% | 2.02% |
| *Bifidobacteriales* | 4.92% | 0.41% | 2.29% | 0.04% | 0.03% | 0.00% |
| *Rhizobiales* | 0.00% | 0.00% | 0.02% | 0.00% | 0.47% | 7.07% |
| *Frankiales* | 0.00% | 0.00% | 0.00% | 0.00% | 1.34% | 4.41% |
| *Campylobacterales* | 0.00% | 0.07% | 0.00% | 0.13% | 5.29% | 0.05% |
| *Chitinophagales* | 0.00% | 0.00% | 0.00% | 0.00% | 0.63% | 4.72% |
| Others (<4%) | 8.45% | 8.92% | 15.51% | 16.65% | 14.02% | 57.96% |

**TABLE S13.** RSD values of sink relative contribution in five independent FEAST runs

| Season | | Sink | Human | Swine | Bovine | Sheep |
| --- | --- | --- | --- | --- | --- | --- |
| Dry | R1 | | **15^a^** | 36 | 18 | 150 |
|  | R2 | | **16** | 33 | 23 | 169 |
|  | R3 | | **7** | 14 | 17 | 224 |
|  | R4 | | **15** | 11 | 26 | 180 |
|  | R5 | | **6** | 26 | 11 | 91 |
|  | R6 | | **8** | 18 | 20 | 75 |
| Wet | R1 | | 31 | 39 | **19** | 45 |
|  | R2 | | 10 | **6** | 12 | 57 |
|  | R3 | | 42 | **14** | 13 | 35 |
|  | R4 | | 27 | **13** | 20 | 80 |
|  | R5 | | 49 | **7** | 7 | 9 |
|  | R6 | | 13 | 18 | **12** | 54 |

^a^ Numbers in bold indicated that the fecal source was calculated as the largest relative contribution in the corresponding sink for total fecal sources.


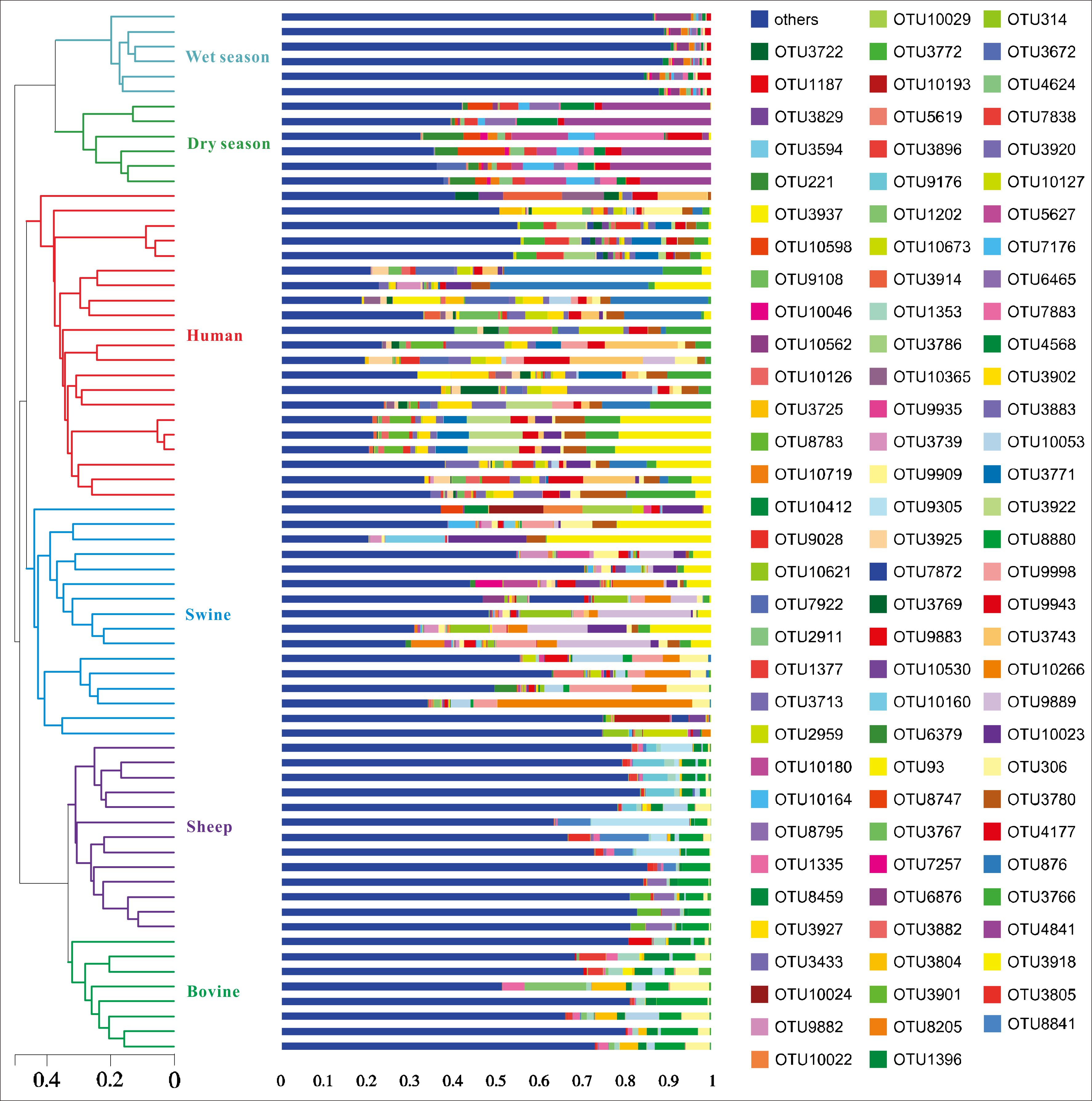


**FIGURE S1.** Hierarchical clustering and taxonomic distribution of taxa in individual water and fecal samples at the OTU level clustered at 97% similarity.


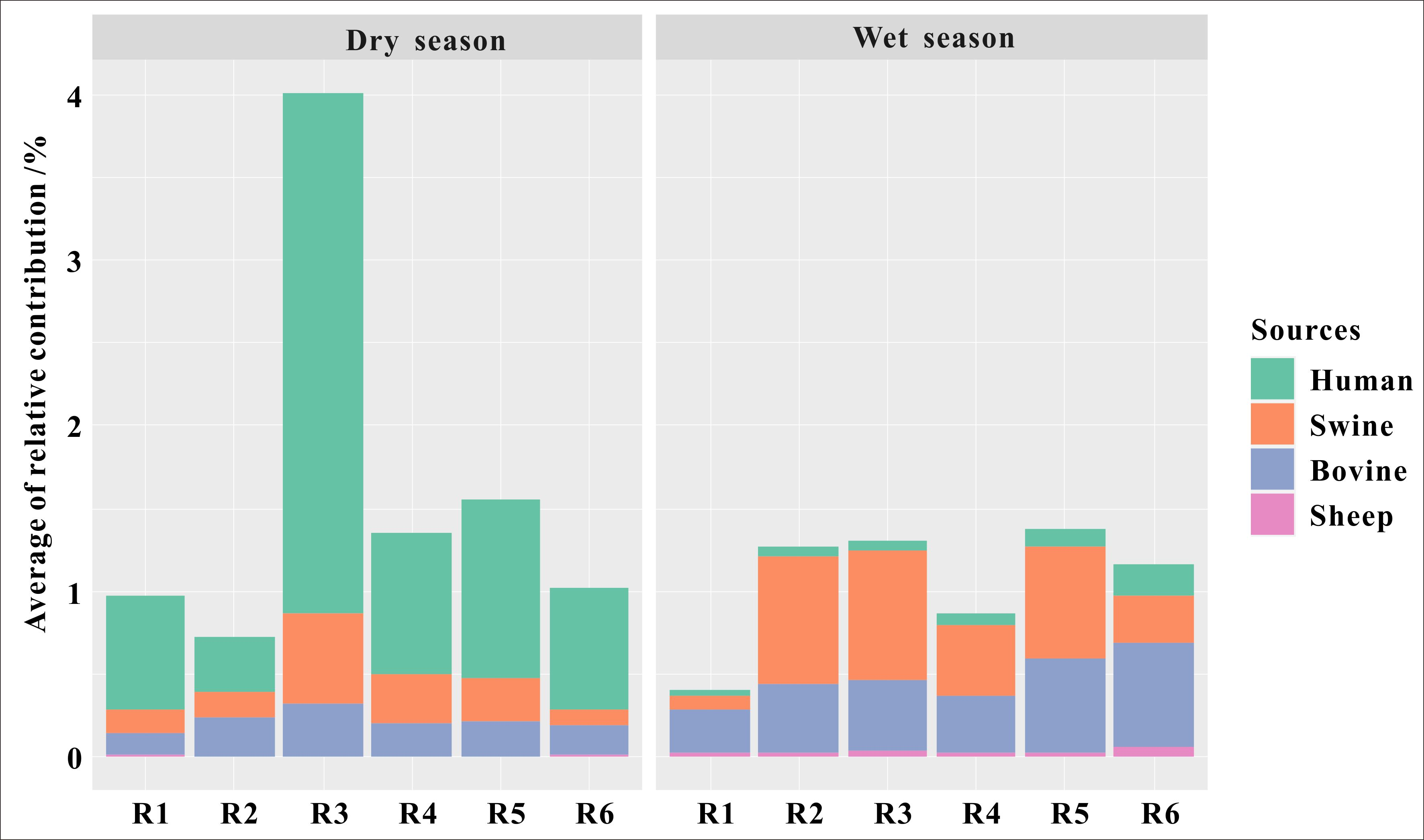


**FIGURE S2** Average relative contribution of sources at river water samples collected from R1-R6 six sites during dry and wet seasons. FEAST was run in five independent runs on the same script.

**REFERENCES**

Bernhard, A. E. and Field, K. G., (2000). A PCR assay to discriminate human and ruminant feces on the basis of host differences in *Bacteroides-Prevotella* genes encoding 16S rRNA. *Appl. Environ. Microbiol.* 66: 4571-4574. doi:10.1128/aem.66.10.4571-4574.2000.

Converse, R. R., Blackwood, A. D., Kirs, M., Griffith, J. F. and Noble, R. T., (2009). Rapid QPCR-based assay for fecal *Bacteroides* spp. as a tool for assessing fecal contamination in recreational waters. *Water Res.* 43: 4828-4837. doi:10.1016/j.watres.2009.06.036.

Green, H. C., Haugland, R. A., Varma, M., Millen, H. T., Borchardt, M. A., Field, K. G. et al., (2014). Improved HF183 quantitative real-time PCR assay for characterization of human fecal pollution in ambient surface water samples. *Appl. Environ. Microbiol.* 80: 3086-3094. doi:10.1128/aem.04137-13.

Kildare, B. J., Leutenegger, C. M., McSwain, B. S., Bambic, D. G., Rajal, V. B. and Wuertz, S., (2007). 16S rRNA-based assays for quantitative detection of universal, human-, cow-, and dog-specific fecal *Bacteroidales*: A Bayesian approach. *Water Res.* 41: 3701-3715. doi:10.1016/j.watres.2007.06.037.

Liang, H. X., Yu, Z. S., Ndayisenga, F., Liu, R. Y., Zhang, Y. M., Zhang, H. X. et al., (2020). A combination of mitochondrial DNA markers Ckmito and ND5-CD is recommended as the most reliable indicator for microbial source tracking to identify faecal pollution from poultry in China. *Ecol. Indic.* 115: 106334. doi:10.1016/j.ecolind.2020.106334.

Mieszkin, S., Furet, J. P., Corthier, G. and Gourmelon, M., (2009). Estimation of pig fecal contamination in a river catchment by real-time PCR using two pig-specific *Bacteroidales* 16S rRNA genetic markers. *Appl. Environ. Microbiol.* 75: 3045-3054. doi:10.1128/aem.02343-08.

Mieszkin, S., Yala, J. F., Joubrel, R. and Gourmelon, M., (2010). Phylogenetic analysis of *Bacteroidales* 16S rRNA gene sequences from human and animal effluents and assessment of ruminant faecal pollution by real-time PCR. *J. Appl. Microbiol.* 108: 974-984. doi:10.1111/j.1365-2672.2009.04499.x.

Ohad, S., Ben-Dor, S., Prilusky, J., Kravitz, V., Dassa, B., Chalifa-Caspi, T. et al., (2016). The development of a novel qPCR assay-set for identifying fecal contamination originating from domestic fowls and waterfowl in Israel. *Front. Microbiol.* 7: 145. doi:10.3389/fmicb.2016.00145.

Reischer, G. H., Ebdon, J. E., Bauer, J. M., Schuster, N., Ahmed, W., Astrom, J. et al., (2013). Performance characteristics of qPCR assays targeting human- and ruminant-associated *Bacteroidetes* for microbial source tracking across sixteen countries on six continents. *Environ. Sci. Technol.* 47: 8548-8556. doi:10.1021/es304367t.

Reischer, G. H., Kasper, D. C., Steinborn, R., Farnleitner, A. H. and Mach, R. L., (2007). A quantitative real-time PCR assay for the highly sensitive and specific detection of human faecal influence in spring water from a large alpine catchment area. *Lett. Appl. Microbiol.* 44: 351-356. doi:10.1111/j.1472-765X.2006.02094.x.

Zhang, Y., Wu, R. R., Lin, K. R., Wang, Y. S. and Lu, J. Q., (2020). Performance of host-associated genetic markers for microbial source tracking in China. *Water Res.* 175: 115670. doi:10.1016/j.watres.2020.115670.
